# Double Trouble: Herpes Simplex Encephalitis Triggering Autoimmune Encephalitis

**DOI:** 10.7759/cureus.80175

**Published:** 2025-03-06

**Authors:** Somarajan Anandan, Parameswaran Krishnan

**Affiliations:** 1 Neurology, St. Joseph's Mission Hospital, Anchal, IND; 2 Neurology, Indo American Brain and Spine Centre, Vaikom, IND

**Keywords:** anti- nmda receptor encephalitis, autoimmune encephalitis, herpes simplex encephalitis (hse), post hse autoimmune encephalitis, post hsv anti-nmda receptor encephalitis

## Abstract

Herpes simplex virus 1 (HSV1) is the most common cause of sporadic encephalitis worldwide. Even with optimal treatment, half of these patients experience significant neurological sequelae. A subset of these patients has recurrent neurological syndromes, occurring within two to three months of disease onset. Often, these patients show no evidence of recurrent herpes simplex virus (HSV) infection. Many develop antibodies against the N-methyl-D-aspartate receptor (NMDAR) . Here, we describe a case of anti-NMDAR encephalitis occurring almost a year after the onset of HSV encephalitis.

## Introduction

Anti-N-methyl-D-aspartate receptor encephalitis (NMDARE) is a serious but treatable autoimmune disorder that presents with a combination of psychiatric and neurological symptoms. Patients commonly experience behavioral changes, psychiatric disturbances, seizures, memory impairment, language dysfunction, involuntary movements, and altered consciousness. Hypoventilation and autonomic instability are often seen in many cases. It is frequently associated with teratomas or other neoplasms, especially in young females [1]. NMDARE has been reported as a complication following herpes simplex virus (HSV) encephalitis, with a seroconversion rate of up to 30%. Symptoms typically emerge within two months of the initial HSV infection and commonly include mood disturbances, psychosis, and seizures [2]. Relapse of HSV occurs in up to 25% of children, often presenting as choreoathetosis [3]. Post-herpes simplex encephalitis acute chorea may result from either resumption of viral replication or an immunoinflammatory response. NMDARE and post-HSV NMDARE may exhibit distinct clinical and radiological features [4].

## Case presentation

**Figure 1:**
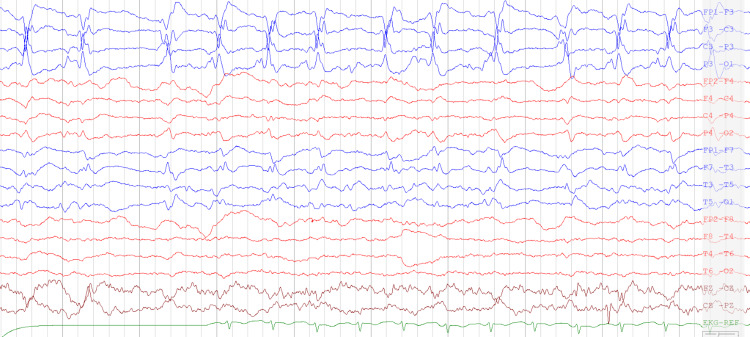
Electroencephalogram showing bilateral lateralized periodic discharges (LPDs) during initial admission.

**Figure 2:**
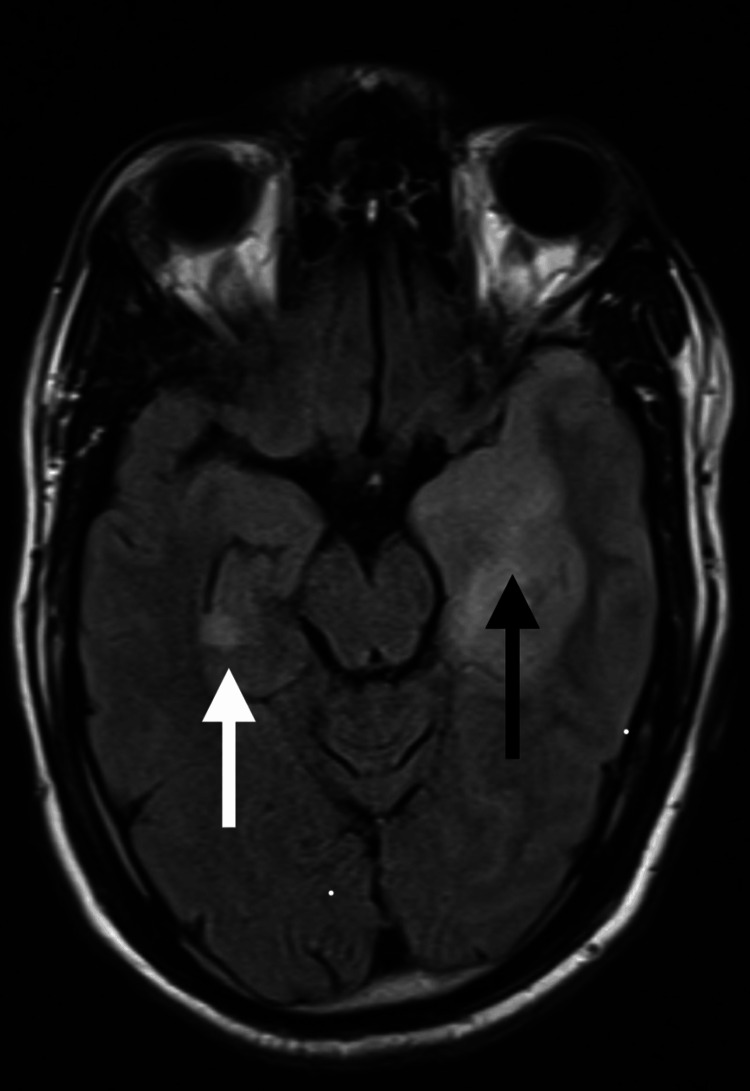
MRI brain axial FLAIR image taken on January 24, 2024, showing left mesial temporal hyperintensity with obliteration of the temporal horn and mild compression of the midbrain (indicated by black arrow) and right hippocampal hyperintensity (indicated by white arrow). FLAIR: Fluid-attenuated inversion recovery.

**Figure 3:**
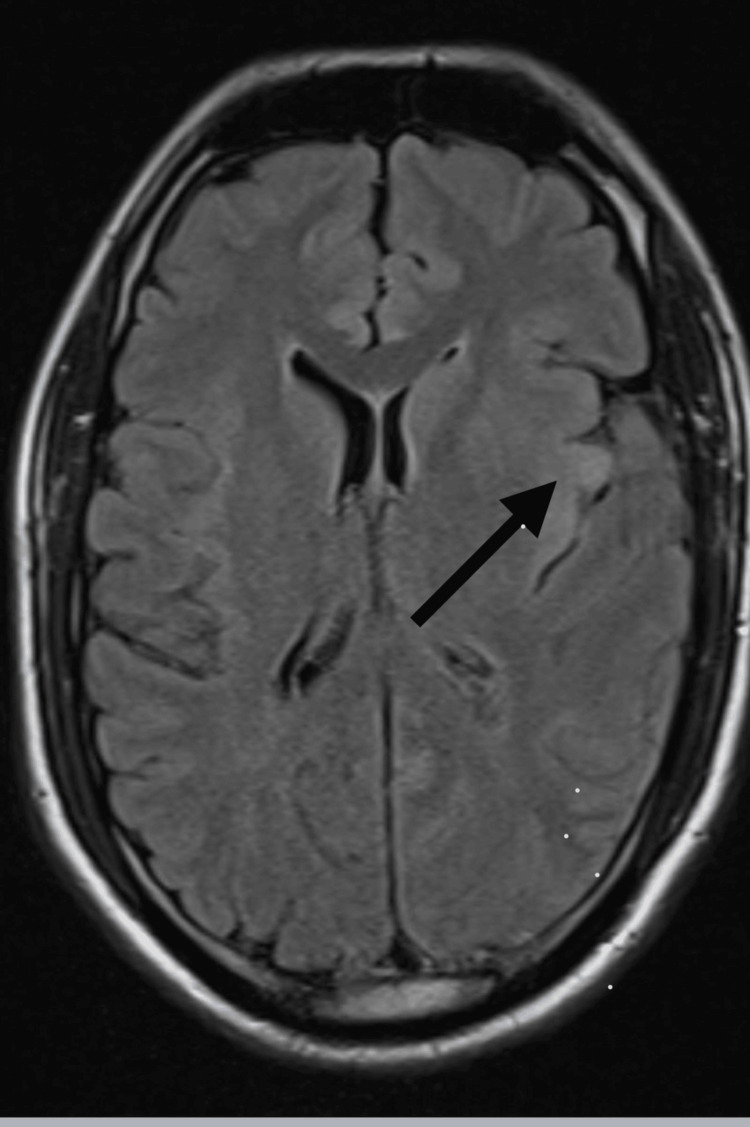
MRI brain axial FLAIR image taken on January 24, 2024, showing left insular hyperintensity with effacement of the left frontal horn (black arrow), and subtle involvement of the lentiform nucleus, frontal operculum, and cingulate gyrus. FLAIR: Fluid-attenuated inversion recovery.

**Figure 4:**
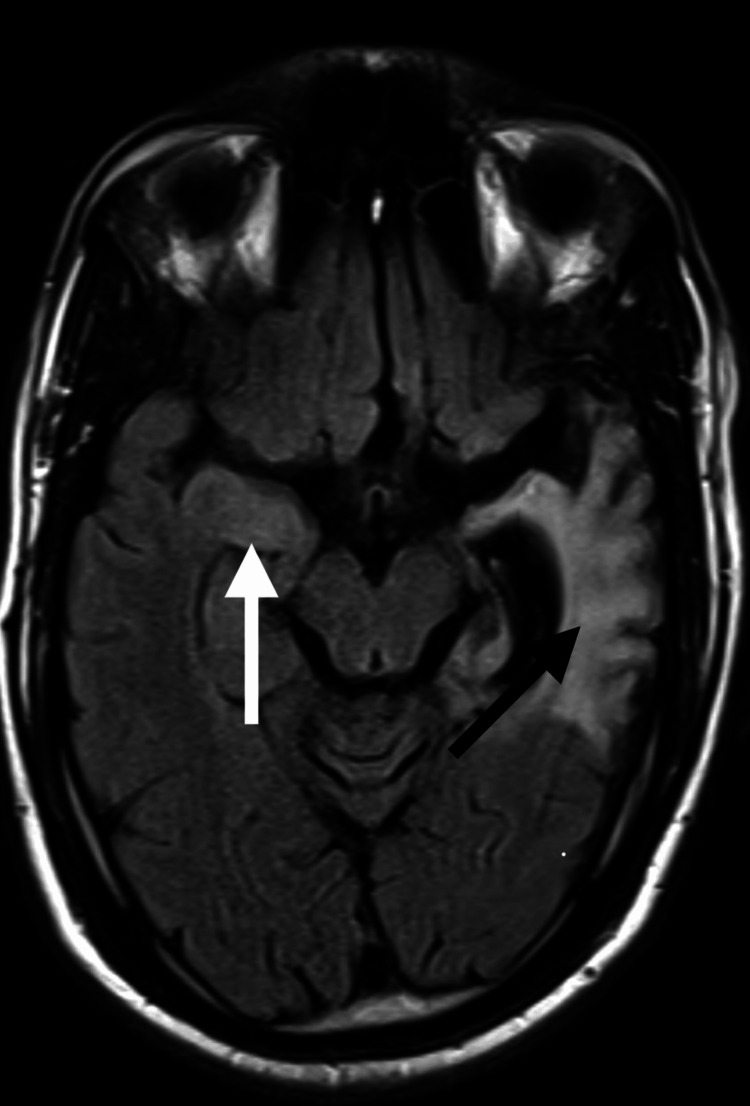
MRI brain axial FLAIR image taken on January 9, 2025, showing left mesial temporal atrophy with ex vacuo hydrocephalus (black arrow) and right mesial temporal hyperintensity (white arrow). FLAIR: Fluid-attenuated inversion recovery.

**Figure 5:**
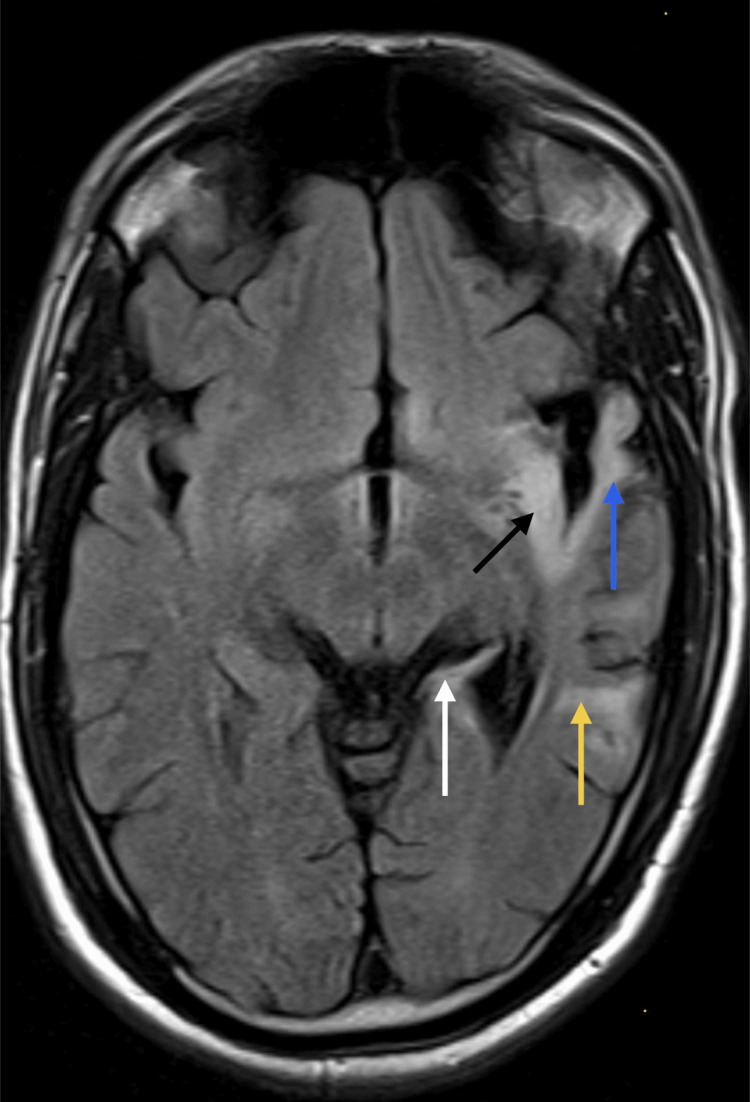
MRI brain axial FLAIR image taken on January 9, 2024, showing hyperintensity involving the left insula (black arrow), temporal operculum (blue arrow), lateral temporal lobe (yellow arrow), posterior hippocampus (white arrow), and bilateral medial orbital cortex. FLAIR: Fluid-attenuated inversion recovery.

A 28-year-old male presented to the neurology clinic with severe holocranial headache associated with vomiting of five days' duration. Three days into his illness, he developed reduced word output with difficulty in naming objects and identifying persons, along with mild memory impairment. There were a few episodes of transient unresponsiveness with orofacial automatisms without limb automatism, lasting 1-2 minutes. There was no history of jerking of limbs or falls. He had a fever at the onset, which persisted for three days. At admission, he had global aphasia. There was no weakness or ataxia. There were no meningeal signs or papilledema. EEG showed bilateral lateralized periodic discharges (LPDs) (Figure 1). His MRI of the brain showed T2/fluid attenuated inversion recovery (FLAIR) hyperintensity in bilateral medial temporal lobes and the left insula (Figures 2-3). His biochemical investigations were normal. Cerebrospinal fluid study showed neutrophilic pleocytosis (240 cells/mm^3^, Neutrophils 90%, Lymphocytes 10%) with normal sugar and protein levels. Herpes simplex type 1 DNA polymerase chain reaction (PCR) was positive, and anti-NMDA receptor antibody was negative. He was treated with intravenous acyclovir 500 mg every 8 hours for 21 days and intravenous levetiracetam 2000 mg per day. He also received dexamethasone 16 mg/day for 7 days. His aphasia recovered, but he had mild episodic memory impairment. After one month, he could return to his duties as a clerk. Almost one year after his HSE, he presented with recurrent episodes of generalized tonic-clonic seizures, a few episodes of focal seizures with impaired awareness, and abnormal behavior in the form of restlessness and irrelevant talking. MRI of the brain showed left temporal atrophy with hydrocephalus ex vacuo changes and right mesial temporal FLAIR hyperintensity (Figures 4-5). There was no contrast enhancement. EEG showed left temporal LPDs. His CSF study showed positive anti-NMDA receptor and HSV DNA PCR was negative (Table 1). He was treated with IV levetiracetam 2000 mg/day and IV methylprednisolone 1 g for five days. His seizures subsided, and behavioral abnormality improved. He was given one dose of injection rituximab. At a follow-up after one month, he is back to his baseline status with mild episodic memory impairment and has rejoined his job. Currently, he is on levetiracetam 2000 mg/day and a tapering dose of steroids. A repeat EEG at follow-up showed rare right temporal sharp waves. We are planning to repeat the CSF anti-NMDA receptor antibody test after six months.

**Table 1:** Cerebrospinal fluid findings. Changes in cerebrospinal fluid during first and second admissions. HSV1: Herpes simplex virus 1; NMDA: N-methyl-D-aspartate.

CSF Findings	January 22, 2024	January 10, 2025	Normal Range
Total count	240 cells/mm³	1 cell/mm³	0-5 cells/mm³
Neutrophils	90%	0%	0%
Lymphocytes	10%	100%	100%
Protein	43 mg%	23 mg%	20-45 mg%
Sugar	72 mg%	77 mg%	50-80 mg%
HSV 1 DNA PCR	Positive	Negative	Negative
NMDA Receptor Antibody (GluN1 subunit)	Negative	Strongly Positive	Negative

## Discussion

The detection of N-methyl-D-aspartate receptor (NMDAR) antibodies in the sera of patients with a history of herpes simplex virus type 1 encephalitis (HSE) was initially an incidental finding in a control group during the development of a standardized NMDAR antibody assay. A subsequent retrospective analysis of 44 HSE patients treated at a German university hospital identified NMDAR antibodies in 30% of these individuals [5]. Similarly, a Spanish study found that 39 out of 93 (42%) patients with HSE developed neuronal autoantibodies, and 21 (54%) of them progressed to autoimmune encephalitis (AE) [6].

Initially, molecular mimicry between HSV surface molecules and the NMDAR was proposed as a potential mechanism. However, this hypothesis was later refuted when NMDAR antibodies were also detected following other viral encephalitides, such as Japanese encephalitis B. The most plausible explanation for the development of post-HSE NMDARE is cross-reactivity induced by the immune response to HSV infection, which exposes normally sequestered central nervous system antigens. Additionally, delayed acyclovir treatment may increase the risk of developing post-HSVE NMDARE, raising discussions about the potential role of corticosteroid therapy in HSVE treatment to prevent autoimmunity [7].

The emerging clinical phenotype of post-HSE AE typically includes a combination of new behavioral changes, encephalopathy, seizures, and movement disorders. Post-HSE AE responds well to immunotherapy (steroids, plasma exchange, intravenous immunoglobulin, and rituximab) and is primarily associated with antibodies targeting synaptic neuronal cell surface receptors, particularly the GluN1 subunit of the NMDAR. Further studies have identified antibodies against other neuroglial targets, including GABA-A receptor, contactin-associated protein-like 2 (CASPR2), leucine-rich glioma-inactivated 1 (LGI1), glial fibrillary acidic protein (GFAP), and dopamine 2 receptor, as well as other unknown antigenic targets [8].

A study found that AE occurred in 27% of patients with HSE. This condition was linked to the development of neuronal antibodies and typically emerged within two months after completing treatment for HSE. The symptoms were age-dependent, with younger children experiencing worse neurological outcomes. Early diagnosis is crucial, as patients, particularly those older than four years, can benefit from immunotherapy [9].

Patients with HSE-associated NMDARE tend to have a poorer long-term prognosis compared to those with primary NMDARE, due to the irreversible neuronal damage caused by the viral infection. Ultimately, post-HSVE AE appears to be more common than previously thought, emphasizing the need to adjust the post-acute management of HSE patients accordingly [10]. A systematic review of the literature revealed that post-HSV anti-NMDAR encephalitis occurs after a median of 24 days in children compared to 40 days in adults [11]. There are very few reports of late presentation anti-NMDAR encephalitis after HSE [12].

## Conclusions

A neurological relapse following primary HSV encephalitis within weeks or months after the initial infection is a well-recognized phenomenon. Relapsing symptoms in post-HSV encephalitis can arise as either a true viral relapse or an immune-mediated disorder. Immune-mediated post-HSV symptoms typically appear within two months of the initial infection.

Post-HSV NMDA encephalitis should be suspected in any patient with a history of herpes encephalitis who develops new-onset behavioral changes, encephalopathy, seizures, or movement disorders. While most cases emerge within two months, post-HSV NMDA encephalitis can manifest up to one year after the initial infection and is responsive to immunotherapy. Our case presented initially as HSE, which was followed by anti-NMDAR encephalitis almost a year after the initial event, despite treatment with steroids in addition to acyclovir.
